# Sacroiliac joint involvement in children with inflammatory bowel diseases

**DOI:** 10.14744/nci.2021.24572

**Published:** 2021-02-08

**Authors:** Nelgin Gerenli, Betul Sozeri, Sevinc Kalin, Heves Kirmizibekmez, Coskun Celtik

**Affiliations:** 1. Department of Pediatric Gastroenterology and Hepatology, University of Health Sciences, Umraniye Training and Research Hospital, Istanbul, Turkey; 2. Department of Pediatric Rheumatology, University of Health Sciences, Umraniye Training and Research Hospital, Istanbul, Turkey; 3. Department of Pediatric Radiology, University of Health Sciences, Umraniye Training and Research Hospital, Istanbul, Turkey; 4. Department of Pediatric Endocrinology, University of Health Sciences, Umraniye Training and Research Hospital, Istanbul, Turkey

**Keywords:** Frequency, pediatric inflammatory bowel diseases, sacroiliitis

## Abstract

**Objective:**

Sacroiliitis (SI), an inflammatory arthropathy, may accompany pediatric inflammatory bowel diseases (IBDs), present with non- specific back pain, hence might be unnoticed. The aims of this study were to assess the frequency of the SI in children with IBD and determine the characteristics of the association of SI with the clinical hallmarks of the IBD.

**Methods:**

In this prospective, cross sectional study, twenty-seven children with IBD, 7–18 years of age were evaluated. Patients with low back pain or stiffness, alternating buttock pain, or hip pain were examined for the presence of SI. The radiologic manifestations on X-ray suggesting sacroilitis were confirmed with Magnetic resonance imaging (MRI).

**Results:**

Twenty-seven children (16 girls, female/male=1.45), with mean age of 12.55±3.6 years, of which 52% had ulcerative colitis (UC), 41% had Crohn’s disease (CD), and two had indeterminate colitis (IC). The median time from IBD diagnosis was 6.0 (18.0) months for patients with SI and 12.0 (13.5) months for patients without SI. Low back pain or stiffness was observed in 13 patients (48%). SI was present in eight (30%) of the children with IBD. The patients with CD were more prone to SI (45% of CD vs. 21% of UC patients). All patients with SI were negative for HLA-B27 genotyping. The disease activity and gender were not associated with increased risk for SI. MRI was remarkable for bone marrow edema in all of the patient, followed by erosions in six of them (75%), synovial enhancement observed in five (63%), and erosion associated enthesitis of the pelvic region was observed in two (25%) of the patients.

**Conclusion:**

SI may remain obscured in children with IBD. Children with CD are more prone to SI than those with UC. Pediatric rheumatology-pediatric gastroenterology collaboration might augment screening in at-risk patients.

**I**nflammatory bowel diseases (IBDs) are a group of chronic inflammatory disorders affecting mainly the gastrointestinal tract. However, approach as a systemic disease should be considered since associated extraintestinal manifestations (EIMs) involving the eyes, skin, hepatobiliary, and musculoskeletal systems may be observed [1]. Musculoskeletal manifestations are the most common EIMs in IBD, found in nearly 20–30% of the patients [2]. Musculoskeletal involvement, affecting the peripheral or axial joints, can precede, concur, or succeed IBD with characteristically pauciarticular, asymmetrical, transitory, migrating, and usually non-deforming lesions [3]. It is well established that the risk of developing peripheral arthritis increases with the extent of colonic disease [4], whereas axial arthropathies are usually not correlated with IBD extent and activity [1]. Axial involvement may vary from asymptomatic sacroiliitis (SI) to ankylosing spondylitis with asymptomatic SI incidence from 10% to 52% in adults with IBD [5].

Previous studies have suggested increased risk for peripheral arthritis and SI in subjects with Crohn’s disease (CD) than those with ulcerative colitis (UC), especially in CD with perianal and upper gastrointestinal involvement [6]. The diagnosis of SI was previously made using plain pelvic radiographs which have low sensitivity and specificity [7, 8]. Magnetic resonance imaging (MRI) can visualize soft tissues changes, and unlike CT, it does not expose the patient to ionizing radiation thus remains the gold standard to detect SIJ involvement in children [9, 10]. The use of MRI in the evaluation of children and adolescents with a clinical suspicion of SI would improve early identification of inflammatory changes, since delayed diagnosis may lead to pain that impairs quality of life, causing debility, and irreversible joint damage [11–13]. Despite growing awareness of the prevalence of SI in IBD, it often goes undiagnosed. This study aimed to investigate the frequency and determine the relation of SI with the clinical hallmarks of the associated IBD.

## Materials and Methods

Twenty-seven children, 7–18 years of age, diagnosed with CD, UC, or indeterminate colitis (IC) followed in a pediatric gastroenterology clinic were included in the study. The patients were receiving 5 ASA and azathioprine treatment, with or without methylprednisolone. Height, weight, and body mass index were settled to age- and sex-adjusted Standard deviation scores (SD Z scores) using World Health Organization’s growth charts. The diagnosis of UC was done if the child with diarrhea and/or blood or mucus in the stools had evidence of continuous macroscopic inflammation on the colonoscopy, extending from the rectum to the proximal regions of the colon and histological features typical of UC, excluding CD of the small bowel through radiology, endoscopy, and histology. CD was diagnosed in the presence of typical clinical features such as weight loss, abdominal pain, and diarrhea with macroscopic inflammation such as skipped and patchy lesions, segmental inflammation, aphtoid ulcers, cobble-stoning or strictures and histological evidence of inflammatory infiltrate toward the lamina propria with or without epithelioid granuloma formation. The diagnosis of IC was done if there were no clear criteria for the diagnosis of CD or UC despite remarkable macroscopic and/or microscopic evidence for the presence of the IBD [14].

**Highlight key points**

• Sacroiliitis in pediatric IBD may be more frequent than expected.
• Early detection and initiation of prompt therapy are essential for preventing permanent joint damage.
• Pediatric rheumatology screening for at-risk patients is needed.

Disease activity indexes for CD and UC were calculated using the Pediatric Cohn’s Disease Activity Index (PCDAI) and the Pediatric Ulcerative Colitis Activity Index (PUCAI) [15, 16]. Clinical information about prior diagnoses and laboratory tests were obtained from the hospital medical record system. Patients who had been previously diagnosed with arthritis, taking non-steroidal anti-inflammatory drugs, or who were unable to describe pain accurately were excluded from the study. All patients were examined for uveitis by a specialized ophthalmologist.

Musculoskeletal examination: All patients, who had musculoskeletal complaints suggestive of SI, were further evaluated by a single pediatric rheumatologist, who was blinded to the specific diagnosis and clinical history of the patient, including the disease subtype, musculoskeletal symptoms, and received therapy. Patients with low back pain or stiffness, alternating buttock pain, or hip pain were further evaluated for the presence of SI. Defined as the painful or asymptomatic inflammation of the sacroiliac joint, SI may present as back pain, usually persisting beyond 3 months, with or without associated morning stiffness which usually improves with physical activity [17]. The sensitivity of tenderness along the sacroiliac joint margin, reduced lumbosacral flexion (Schober test), or a positive Patrick’s test (or FABER test, for pain elicited by flexion, abduction, and external rotation of the leg) for predicting sacroiliac joint inflammation is supposed to be relatively low [17, 18]. The patients with SI were also questioned and examined for the other signs suggesting spondiloarthropathies as dactilitis, inflammatory pain and enthesitis.

The radiologic manifestations on X-ray suggesting sacroilitis were confirmed with MRI [10, 11]. All MR images were obtained with a 1.5T (Siemens Magnetom Aera Healthcare, Erlangen, Germany) with a standard body coil. The standard protocol included coronal oblique short tau inversion recovery, coronal oblique T1-weighted spin-echo non-fat-saturated, T1-weighted spin-echo fat-saturated pre and post-contrast images in axial section. The most relevant manifestation of SI on MR images was the presence of bone marrow edema (BME) of the subchondral regions with peri-articular high T2-weighted signal with low T1-weighted signal. The supporting findings were synovial enhancement, synovial effusions, sclerosis, and erosions [11]. Abnormal enhancement of the subchondral or periarticular regions or synovitis with an enhancement of the synovium was specific for osteitis [12]. The effusion was defined as fluid/high T2-weighted signal within the sacroiliac joint. The erosion was described as irregular indentations of the articular surface of the sacroiliac joint, with corresponding low T1-weighted signal, with low or high T2-weighted signal depending on the presence of active inflammation. The high T2-weighted signal intensity or enhancement of the capsule, insertions of ligaments or tendons represented capsulitis or enthesitis, respectively, subchondral sclerosis was considered if there were a low T1- and T2-weighted signal bands in the periarticular region while ankylosis is suspected in the presence of periarticular low signal on all sequences with blurring of the joint margins [11, 12].

Local Ethics Committee has approved the study (approval date: 13.08.2020, number: B.10.1.TKH.4.34.H.GP.O.01/292).

### Statistical Analysis

The data were analyzed by the Statistical Package for the Social Sciences (SPSS) version 11 for Windows (SPSS Inc.; Chicago, IL, USA). Shapiro-Wilks test was used to determine the distribution of the variables. Independent Samples t-test was used for comparison of normally distributed data; accordingly the results were shown as mean and SD. Mann Whitney U test was used for non-normally distributed variables and the results were reported as interquartile range. Pearson Correlation Test, Fisher’s exact test, and Chi-square test were used to compare the absence or presence of SI, and p<0.05 were accepted as significant.

## Results

The trial involved 27 children (16 girls, female/male = 1.45), with a mean age of 12.55 ± 3.6 years, of which fourteen (52%) had UC, eleven (41%) had CD, and two (7%) had IC. The median time from the diagnosis of IBD was 6.0 (18.0) months in patients with SI and 12.0 (13.5) months in patients without SI. A total of 13 patients (48%) with symptomatic low back pain and/or morning stiffness were examined and SI was found in eight (30%) of them. Bilateral SI was observed in three of the patients, two with CD, one with UC, whereas five had only unilateral involvement (three with CD, two with UC). Disease phenotype and medications for IBH treatment were summarized in Table 1.

**Table 1.** Characteristics of patients with IBD according to sacroiliac joint involvement, disease location and medications

Disease type and number of patients	All	UC	CD	IC
	(27)	(14)	(11)	(2)
Low back pain n (13)		5	7	1
Sacroiliitis n (8)		3	5	0
Disease location, n				
Rectosigmoid		3		1
Left sided disease		2		1
Pancolitis		9		
Large intestine only			2	
Small and large intestine			9	
Medications, (%)				
5-Acetylsalicylic acid	100	52	41	7
Azathioprine	85	44	41	0
Methylprednisolone	19	4	15	0

IBD: Inflammatory bowel disease; CD: Crohn’s disease; UC: Ulcerative colitis; IC: Indeterminate colitis.

The association between SI and clinical variables, including age, gender, body mass index SDs, IBD activity index, and SI distribution among IBD type were detailed in Table 2. The results of blood tests obtained at the time of examination were also summarized in Table 2.

**Table 2.** Demographic, biochemical and clinical characteristics of IBD patients with and without sacroiliitis

Sacroiliitis	Present (n=8)	Absent (n=19)	p
Gender (F/M)	4/4	12/7	0.824
Age (years), mean±SD	11.8±3.7	13.3±3.5	0.182
Weight SDS, mean±SD	0.19±0.95	-0.18±1.23	0.453
BMI SDS, mean±SD	-0.27±0.63	-0.11±1.25	0.737
Hemoglobin (g/dl), mean±SD	12.2±0.74	12.6±0.93	0.240
WBC (×10^3^/μL), mean±SD	9.55 (4.52)	8.90 (3.33)	1.00
Platelet (×10^3^/μL), mean±SD	380±84	359±296	0.465
Albumin (g/dl), mean±SD	3.91±0.24	3.97±0.25	0.569
ESR (mm/h), median (IQR)	28 (24)	22 (11)	0.038
CRP (mg/dl), median (IQR)	0.55 (0.48)	0.20 (0.20)	0.022
Duration of disease (months), median (IQR)	6.0 (18.0)	12.0 (13.5)	0.391
PCDAI, median (IQR)	28.0 (25.0)	30.0 (5.0)	0.452
PUCAI, median (IQR)	24.0 (1.25)	20.0 (10.0)	0.229
IBD activity-remission, (%)	4	29	0.778
IBD activity-mild to moderate disease, (%)	26	41

SD: Standard deviation; Weight SDS: Weight standard deviation score; BMI SDS: Body mass index standard deviation score; IQR: Interquartile range; IBD: Inflammatory bowel disease; IC: Indeterminate colitis; PCDAI: Pediatric crohn’s disease activity index; PUCAI: Pediatric ulcerative colitis activity index; ESR: Erythrocyte sedimentation rate; CRP: C-reactive protein; WBC: White blood cell.

All patients with SI were negative for HLA-B27genotyping and had no perinuclear anti-neutrophil cytoplasmic antibodies; however, two of them were positive for anti-nuclear antibodies (at levels of 1/40 and 1/80, respectively). Erythema nodosum was previously diagnosed in three subjects (9%), two with UC and one with IC; however, none of these patients had SI. No dactilitis was observed, however, concomitant enthesial involvement was noted in six of the patients with SI.

Ophthalmic examination revealed no uveitis. Differences between UC and CD according to SIJ involvement were detailed in Table 3.

**Table 3.** Features of the sacroileitis in patients with Crohn’s disease or ulcerative colitis

	Crohn’s disease (n=11)	Ulcerative colitis (n=14)	p
Age (years) mean (±SD)	12.5±0.74	13.5±3.5	0.335
Gender (F/M)	5/6	9/5	0.479
Sacroiliitis present (%)	45	21	0.00
Bilateral sacroiliitis (n)	2	2	
Unilateral sacroiliitis (n)	3	1	

SD: Standard deviation; F: Female; M: Male; N: Number.

On MRI, BME was detected in all patients with SI (100%) (affecting the sacral side in two, iliac side in three, and both sides in three), followed by erosions in six patients (75%) (right-sided in two [both on the ilac side] and left-sided in four patients [two on the ilaic and two on the sacral side]), synovial enhancement in five (63%) and erosion-associated enthesitis of the pelvic region in two (25%) of the patients. No subchondral sclerosis or ancylosis was noted. Children, diagnosed with SI were further treated with sulphasalazine (three patients with UC) alone or azathioprine was switched to methotrexate (two patients with CD) and intravenous infusions of anti-TNF monoclonal antibody.

## Discussion

IBD-associated SI was detected in eight out of 27 patients with IBD (30%). As far as we know, this is the first pediatric rheumatology-pediatric gastroenterology collaborated study, reporting SI in children with IBD, based upon the physical examination findings, confirmed by MRI. Recent studies showed a close relationship between IBD and SpAs. The common pathogenic pathway for both entities may explain this association [5]. AS and SI are estimated to occur in about 2–16% and 12–46% of IBD patients, respectively [4, 6, 19]. In a study performed in adults with IBD and based upon physical examination and lumbosacral X-ray, subclinical SI was found in 24% of the patients [20]. As outlined in the present study nearly 30% of the children with IBD had SI. This observation is in accordance with the previous study, reporting EIMs of IBD, where SI in pediatric IBD patients was found to be 33% (26 of 78 patients) [7]. Nevertheless, patients’ presenting symptoms may also play a role in detecting joint involvements. In the present study, half of the patients diagnosed with SI were presented to the pediatric rheumatology unit and thereafter received IBD diagnosis. One can speculate that SI involvement was observed more frequently compared to the literature due to this reason. Levy et al. [21] screened children presented with musculoskeletal symptoms and observed sacroiliac involvement in 34.8% of them and these children later received IBD diagnosis, whereas SI was observed only in 2.2% of the controls. Of note, EIMs occurred at lower frequency in elderly-onset compared with pediatric-onset IBD patients [22]. Hwangbo et al. [23] evaluated abdominopelvic tomography of the IBD patients and showed that SI was more prevalent among young patients with up to 40.0% in the ≤18 years of age group. A recent meta-analysis regarding mean age showed that the prevalence of SI seemed to be highest in the young group (16% in 20–30 years age category) and decreased to 9% in the 30–40 years group [6]. This finding may explain the higher rates of SI in pediatric trials.

Interestingly SI, diagnosed by symptoms or imaging was more common in females than males within the pediatric IBD group (46.15% vs. 20.0%) [21]. Accordingly, Fauny et al. [24], showed that SI is three times more often in females with low back pain and IBD. Lakatos et al. [25] reported that the frequency of arthropathy is increased in females with CD (20%) compared to UC (15%), especially in children. However, in another pediatric study based solely on MR findings, SI was detected only in males with IBD (5 males, 4 with CD) [26]. In a recent study SI was observed in 63.3% of adult males with IBD [16], whereas no gender difference was shown in the study performed on Korean adult patients with CD and SI [23]. This current study also showed no gender differences among IBD patients with SI.

The EIMs of the IBD’s were more frequently observed in children with CD compared to UC or IC (22.5% vs. 10.3%) [27]. Accordingly, SpA (axial or peripheral) is more commonly observed in patients with CD than those with UC [3, 27]. Kelly et al. [19] showed that 63.3% of the IBD patients with SI had CD, similarly another adult study found the prevalence of SI to be 12.2% in the patients with UC and 21.0% in those with CD [23]. A recent meta-analysis performed by Karreman et al. [6] revealed the prevalence of SI to be 13% and 7% in patients with CD and UC, respectively. The current study correlated with previous trials showing a frequency of CD in 63% of patients with SI.

There is a controversy about the relationship of SI and the duration of IBD. In adults, there was no association between SI and disease duration [5, 19]. Accordingly many studies revealed no relation with duration of the IBD and SI [3, 6, 7]. However, the “duration of disease” was the major limitation in most of them, including our study with a duration of disease of 6 months in patients with SI and 12 months in ones with no SI [3, 6, 7, 23]. Further long-term pediatric studies are needed to evaluate the effect of the duration of IBD on the development of SI.

One of the interesting findings of the present study was that patients with SI had elevated erythrocyte sedimentation rate and C-reactive protein levels; however, PCDAI and PUCAI scores are similar with no differences in aspect of disease activity between the two groups. Queiro et al. found no relationship between SI and the type or severity of IBD, such as Kelly et al. who also had found no significant correlation with any measure of SI and the disease activity [19, 20]. However, Nir et al. [28], reported that patients with peripheral articular involvement had more severe IBD than without articular symptoms. The current study demonstrated that the duration of the IBD was shorter in children with SI, therefore the increased levels of acute-phase reactants in these patients were observed when IBD disease control was not achieved. One can speculate that SI may be more commonly observed at newly diagnosed IBD children.

No one of our patients with SI had HLA-B27 concurrence; however there exists a well-known association between the presence of HLA-B27 and SpAs [29], suggesting that nearly all IBD patients who are positive for HLAB27 will develop AS [5]. Conversely, it has been proposed that patients with SpA, especially with SI and negative HLA B27, are at an increased risk for developing IBD than patients with HLA-B27 positive SpA [13, 30]. In a pediatric study, evaluating chronic intestinal inflammation in seronegative SpAs, all patients with SpA and IBD had negative HLA B27 status [7]. Previous studies demonstrated that patients with IBD-related AS and IBD-related isolated SI are HLA-B27 positive in about 25%–78% and 7%–15%, respectively, proposing that isolated SI is of different nature compared to AS in the setting of IBD [7, 29, 31]. We observed unilateral SI in 63% of IBD patients with SI, however, it is known that SI might be unilateral in the early phase of the disease and may progress to bilateral SI in time [28]. Although none of the patients fulfilled the criteria for AS, we agreed that prompt and long-term follow-up is needed.

As noted in previous trials, as well as the present study, bone marrow edema and erosions were the most common findings of SI in children [32]. Structural changes such as sclerosis were not observed, as these manifestations are uncommon in children [32]. The MRI findings of SI without standardization are problematic, moreover normal MRI appearances of the immature skeleton are not well known [11, 32]. However, with the use of MRI, the frequency of SI diagnosis in patients with IBD increased to rates of as high as 46% [19]. Moreover Queiro et al. [20] detected subclinical low-grade SI in the 60% of the patients with IBD who underwent MR study. The use of MRI in the evaluation of children with a clinical suspicion of SI might improve early diagnosis, identification of inflammatory changes, and treatment [11, 33]. Accordingly, we also detected abnormalities on the MR images in patients suspected to have SI. Children, suspected to have SI were further evaluated and switch to another immunomodulator (methotrexate) or biological therapy (infliximab) was considered [33].

The present study had a few limitations. The study was done with a limited number of patients; therefore it can be accepted as preliminary trial. Moreover, four of the patients with SI presented initially to the pediatric rheumatology unit, IBD diagnosis were received thereafter; hence the presence of the rheumatology unit may contribute to higher detection rates of the SI. In addition, many of the drugs used to control IBD, including salicylate derivatives (used by all of our patients), cytotoxic medications such as azathioprine (used by 85% of the patients) and oral steroids (used by 19%), are also effective in the treatment of SI. This may interfere with the musculoskeletal pain perception as well as with timed diagnosis of SI, therefore naïve patients with active intestinal disease and drug-free may have increased SI frequencies. Two or more SIJ examinations, first at the diagnosis and thereafter, at the time of remission, would give more extensive information about the IBD associated SI in children.

### Conclusion

In conclusion, this analysis of the children with IBD found a high prevalence of SI, especially in patients with CD which was consistent with previous adult and pediatric studies. Children with IBD need to be carefully screened for the presence of back pain as part of their routine follow-up. Of note, depending on the leading manifestations, patients are followed up by a gastroenterologist or a rheumatologist that might overlook the articular or bowel manifestations of the disease, respectively. Delayed diagnosis and inappropriate treatment such as immobilization can contribute significantly to morbidity such as irreversible joint damage.

## References

[1] Rothfuss KS, Stange EF, Herrlinger KR (2006). Extraintestinal manifestations and complications in inflammatory bowel diseases.. World J Gastroenterol.

[2] Bernstein CN, Wajda A, Blanchard JF (2005). The clustering of other chronic inflammatory diseases in inflammatory bowel disease: a population-based study.. Gastroenterology.

[3] Dotson JL, Hyams JS, Markowitz J, LeLeiko NS, Mack DR, Evans JS (2010). Extraintestinal manifestations of pediatric inflammatory bowel disease and their relation to disease type and severity.. J Pediatr Gastroenterol Nutr.

[4] Sheth T, Pitchumoni CS, Das KM (2014). Musculoskeletal manifestations in inflammatory bowel disease: a revisit in search of immunopathophysiological mechanisms.. J Clin Gastroenterol.

[5] Fragoulis GE, Liava C, Daoussis D, Akriviadis E, Garyfallos A, Dimitroulas T (2019). Inflammatory bowel diseases and spondyloarthropathies: From pathogenesis to treatment.. World J Gastroenterol.

[6] Karreman MC, Luime JJ, Hazes JMW, Weel AEAM (2017). The Prevalence and Incidence of Axial and Peripheral Spondyloarthritis in Inflammatory Bowel Disease: A Systematic Review and Meta-analysis.. J Crohns Colitis.

[7] Conti F, Borrelli O, Anania C, Marocchi E, Romeo EF, Paganelli M (2005). Chronic intestinal inflammation and seronegative spondyloarthropathy in children.. Dig Liver Dis.

[8] Weiss PF, Xiao R, Brandon TG, Biko DM, Maksymowych WP, Lambert RG (2018). Radiographs in screening for sacroiliitis in children: what is the value?. Arthritis Res Ther.

[9] Chan J, Sari I, Salonen D, Silverberg MS, Haroon N, Inman RD (2018). Prevalence of sacroiliitis in inflammatory bowel disease using a standardized computed tomography scoring system.. Arthritis Care Res (Hoboken).

[10] de Hooge M, van den Berg R, Navarro-Compán V, van Gaalen F, van der Heijde D, Huizinga T (2013). Magnetic resonance imaging of the sacroiliac joints in the early detection of spondyloarthritis: no added value of gadolinium compared with short tau inversion recovery sequence.. Rheumatology (Oxford).

[11] Weiss PF, Brandon TG, Bohnsack J, Heshin-Bekenstein M, Francavilla ML, Jaremko JL (2021). Variability in interpretation of magnetic resonance imaging of the pediatric sacroiliac joint.. Arthritis Care Res (Hoboken).

[12] Madsen KB, Egund N, Jurik AG (2010). Grading of inflammatory disease activity in the sacroiliac joints with magnetic resonance imaging: comparison between short-tau inversion recovery and gadolinium contrast-enhanced sequences.. J Rheumatol.

[13] Ossum AM, Palm Ø, Lunder AK, Cvancarova M, Banitalebi H, Negård A, IBSEN Study Group (2018). Ankylosing spondylitis and axial spondyloarthritis in patients with long-term inflammatory bowel disease: results from 20 years of follow-up in the IBSEN study.. J Crohns Colitis.

[14] Levine A, Griffiths A, Markowitz J, Wilson DC, Turner D, Russell RK (2011). Pediatric modification of the Montreal classification for inflammatory bowel disease: the Paris classification.. Inflamm Bowel Dis.

[15] Loonen HJ, Griffiths AM, Merkus MP, Derkx HH (2003). A critical assessment of items on the Pediatric Crohn’s Disease Activity Index.. J Pediatr Gastroenterol Nutr.

[16] Turner D, Hyams J, Markowitz J, Lerer T, Mack DR, Evans J, Pediatric IBD Collaborative Research Group (2009). Appraisal of the pediatric ulcerative colitis activity index (PUCAI).. Inflamm Bowel Dis.

[17] Ringold S, Angeles-Han ST, Beukelman T, Lovell D, Cuello CA, Becker ML (2019). 2019 American College of Rheumatology/Arthritis Foundation Guideline for the treatment of juvenile idiopathic arthritis: therapeutic approaches for non-systemic polyarthritis, sacroiliitis, and enthesitis.. Arthritis Care Res (Hoboken).

[18] Ramanathan A, Srinivasalu H, Colbert RA (2013). Update on juvenile spondyloarthritis.. Rheum Dis Clin North Am.

[19] Kelly OB, Li N, Smith M, Chan J, Inman RD, Silverberg MS (2019). The prevalence and clinical associations of subclinical sacroiliitis in inflammatory bowel disease.. Inflamm Bowel Dis.

[20] Queiro R, Maiz O, Intxausti J, de Dios JR, Belzunegui J, González C (2000). Subclinical sacroiliitis in inflammatory bowel disease: a clinical and follow-up study.. Clin Rheumatol.

[21] Levy R, Amarilyo G, Tal R, Amir J, Assa A, Rinawi F (2019). Musculoskeletal manifestations as presenting symptoms of inflammatory bowel disease in children and adolescents.. J Pediatr.

[22] Duricova D, Sarter H, Savoye G, Leroyer A, Pariente B, Armengol-Debeir L, Epimad Group (2019). Impact of extra-intestinal manifestations at diagnosis on disease outcome in pediatric- and elderly-onset Crohn’s disease: A French population-based study.. Inflamm Bowel Dis.

[23] Hwangbo Y, Kim HJ, Park JS, Ryu KN, Kim NH, Shim J (2010). Sacroiliitis is common in Crohn’s disease patients with perianal or upper gastrointestinal involvement.. Gut Liver.

[24] Fauny M, Cohen N, Morizot C, Leclerc-Jacob S, Wendling D, Lux G (2020). Low back pain and sacroiliitis on cross-sectional abdominal imaging for axial spondyloarthritis diagnosis in inflammatory bowel diseases.. Inflamm Intest Dis.

[25] Lakatos L, Pandur T, David G, Balogh Z, Kuronya P, Tollas A (2003). Association of extraintestinal manifestations of inflammatory bowel disease in a province of western Hungary with disease phenotype: results of a 25-year follow-up study.. World J Gastroenterol.

[26] Giani T, Bernardini A, Basile M, Di Maurizo M, Perrone A, Renzo S (2020). Usefulness of magnetic resonance enterography in detecting signs of sacroiliitis in young patients with inflammatory bowel disease.. Pediatr Rheumatol Online J.

[27] Greuter T, Bertoldo F, Rechner R, Straumann A, Biedermann L, Zeitz J, Swiss IBD Cohort Study Group (2017). Extraintestinal manifestations of pediatric inflammatory bowel disease: prevalence, presentation, and anti-TNF treatment. J Pediatr Gastroenterol Nutr.

[28] Nir O, Rinawi F, Amarilyo G, Harel L, Shamir R, Assa A (2017). Phenotypic features and longterm outcomes of pediatric inflammatory bowel disease patients with arthritis and arthralgia.. J Rheumatol.

[29] Orchard TR, Holt H, Bradbury L, Hammersma J, McNally E, Jewell DP (2009). The prevalence, clinical features and association of HLA-B27 in sacroiliitis associated with established Crohn’s disease.. Aliment Pharmacol Ther.

[30] Baeten D, De Keyser F, Mielants H, Veys EM (2002). Ankylosing spondylitis and bowel disease.. Best Pract Res Clin Rheumatol.

[31] Olivieri I, Cantini F, Castiglione F, Felice C, Gionchetti P, Orlando A (2014). Italian Expert Panel on the management of patients with coexisting spondyloarthritis and inflammatory bowel disease.. Autoimmun Rev.

[32] Orr KE, Andronikou S, Bramham MJ, Holjar-Erlic I, Menegotto F, Ramanan AV (2018). Magnetic resonance imaging of sacroiliitis in children: frequency of findings and interobserver reliability.. Pediatr Radiol.

[33] Jaremko JL, Liu L, Winn NJ, Ellsworth JE, Lambert RG (2014). Diagnostic utility of magnetic resonance imaging and radiography in juvenile spondyloarthritis: evaluation of the sacroiliac joints in controls and affected subjects.. J Rheumatol.

